# Disrupted‐in‐schizophrenia‐1 protects synaptic plasticity in a transgenic mouse model of Alzheimer’s disease as a mitophagy receptor

**DOI:** 10.1111/acel.12860

**Published:** 2018-11-28

**Authors:** Zhao‐Tao Wang, Mei‐Hong Lu, Yan Zhang, Wen‐Li Ji, Lei Lei, Wang Wang, Li‐Pao Fang, Lu‐Wen Wang, Fan Yu, Ji Wang, Zhen‐Yu Li, Jian‐Rong Wang, Ting‐Hua Wang, Fei Dou, Qin‐Wen Wang, Xing‐Long Wang, Shao Li, Quan‐Hong Ma, Ru‐Xiang Xu

**Affiliations:** ^1^ Department of Neurosurgery Affiliated Bayi Brain Hospital, General Army Hospital Southern Medical University Beijing China; ^2^ Jiangsu Key Laboratory of Neuropsychiatric Diseases, Institute of Neuroscience Soochow University Suzhou China; ^3^ Department of Physiology, Liaoning Provincial Key Laboratory of Cerebral Diseases Dalian Medical University Dalian China; ^4^ Department of Pathology Case Western Reserve University Cleveland Ohio; ^5^ Hematology Center of Cyrus Tang Medical Institute, Collaborative Innovation Center of Hematology, Key Laboratory of Stem Cells and Biomedical Materials of Jiangsu Province and Chinese Ministry of Science and Technology, State Key Laboratory of Radiation Medicine and Radioprotection Soochow University School of Medicine Suzhou China; ^6^ Institute of Neuroscience Kunming Medical University Kunming China; ^7^ College of Life Sciences Beijing Normal University Beijing China; ^8^ Ningbo Key Laboratory of Behavioral Neuroscience, Zhejiang Provincial Key Laboratory of Pathophysiology, School of Medicine Ningbo University Ningbo China

**Keywords:** Alzheimer’s disease, autophagy, Disrupted‐in‐schizophrenia‐1, mitochondria, mitophagy

## Abstract

Mitochondrial dysfunction is an early feature of Alzheimer's disease (AD). Accumulated damaged mitochondria, which are associated with impaired mitophagy, contribute to neurodegeneration in AD. We show levels of Disrupted‐in‐schizophrenia‐1 (DISC1), which is genetically associated with psychiatric disorders and AD, decrease in the brains of AD patients and transgenic model mice and in Aβ‐treated cultured cells. Disrupted‐in‐schizophrenia‐1 contains a canonical LC3‐interacting region (LIR) motif (^210^FSFI^213^), through which DISC1 directly binds to LC3‐I/II. Overexpression of DISC1 enhances mitophagy through its binding to LC3, whereas knocking‐down of DISC1 blocks Aβ‐induced mitophagy. We further observe overexpression of DISC1, but not its mutant (muFSFI) which abolishes the interaction of DISC1 with LC3, rescues Aβ‐induced mitochondrial dysfunction, loss of spines, suppressed long‐term potentiation (LTP). Overexpression of DISC1 via adeno‐associated virus (serotype 8, AAV8) in the hippocampus of 8‐month‐old APP/PS1 transgenic mice for 4 months rescues cognitive deficits, synaptic loss, and Aβ plaque accumulation, in a way dependent on the interaction of DISC1 with LC3. These results indicate that DISC1 is a novel mitophagy receptor, which protects synaptic plasticity from Aβ accumulation‐induced toxicity through promoting mitophagy.

## 1 INTRODUCTION

Alzheimer's disease (AD), one of the most common neurodegenerative diseases, is characterized by amyloid plaques formed by accumulation of amyloid‐β (Aβ) and neurofibrillary tangles formed by hyperphosphorylated tau (Sun et al., 2018). Mitochondrial abnormalities have been observed in the brains of AD patients, which are even prior to the onset of histopathological or clinical features (Gibson & Shi, 2010; Kerr et al., 2017). Dysfunctional mitochondria result in reduced cellular ATP levels and excessive reactive oxygen species (ROS) production, which leads to Aβ generation and tau phosphorylation. The latter in turn exacerbates mitochondria damage (Kerr et al., 2017). These damaged mitochondria accumulate in AD neurons that are thought to contribute to neurodegeneration with unknown molecular mechanisms (Du, Guo, & Yan, 2012; Reddy, 2013).

Mitophagy, a selective autophagy for the removal of dysfunctional mitochondria, is a key pathway in mitochondrial quality control. The damaged mitochondria are selectively gulfed into autophagosomes and subsequently degraded within lysosomes which are fused with autophagosomes (Liu, Sakakibara, Chen, & Okamoto, 2014). Mitophagy is comprised of AD that contributes to mitochondrial dysfunction in AD (Kerr et al., 2017). The selectivity is conveyed by mitophagy receptors, which localize in mitochondria and contain LC3‐interacting region (LIR) motifs, characterized with “W/F/Y‐x‐x‐L/I/V” surrounded by acidic charged amino acids. Through the LIR motifs, the receptors bind to LC3 and recruit the assembling autophagosomes to the damaged mitochondria (Liu et al., 2014). Several mitophagy receptors such as p62, NBR1, Nix, Tax1BP1, NDP51, optineurin, Ambra1, and FUNDC1 have been identified so far (He, Chen, & Li, 2017; Lazarou et al., 2015; Liu et al., 2012; Strappazzon et al., 2015). Among them, FUNDC1 is involved in hypoxia‐induced mitophagy (Liu et al., 2012). Mutations in optineurin leading to amyotrophic lateral sclerosis (ALS) result in impaired mitophagy (Lazarou et al., 2015). However, it remains unknown how mitophagy is linked to cognitive deficits in AD, and whether other novel mitophagy receptors exist, which are closely linked to AD.

Disrupted‐in‐schizophrenia‐1 (DISC1) is a genetic risk factor for various psychiatric disorders including schizophrenia, major depression, and bipolar disorders (Thomson et al., 2013). Disrupted‐in‐schizophrenia‐1 has many isoforms in human brains, around 50 or more, which are developmentally regulated, suggesting that splice variants are likely to have distinct functions (Nakata et al., 2009). Disrupted‐in‐schizophrenia‐1 regulates a broad of biological and cellular processes such as axonal transport, neuronal development, differentiation, and proliferation of neural stem cells, synaptic functions (Bradshaw & Porteous, 2012; Dahoun, Trossbach, Brandon, Korth, & Howes, 2017; Devine, Norkett, & Kittler, 2016; Lipina & Roder, 2014; Randall, Kurihara, Brandon, & Brown, 2014; Tomoda, Hikida, & Sakurai, 2017). Disrupted‐in‐schizophrenia‐1 exhibits such diverse cellular functions via its interaction with distinct proteins (Bradshaw & Porteous, 2012; Lipina & Roder, 2014; Randall et al., 2014). Recent studies indicate a close link of DISC1 to AD pathogenesis. A genome‐wide association study indicates that DISC1 genetically associates with late onset AD (Beecham et al., 2009). Disrupted‐in‐schizophrenia‐1 regulates Aβ generation through modulating intracellular trafficking of APP (Shahani et al., 2015). Disrupted‐in‐schizophrenia‐1 promotes BACE1 to translocate to the lysosomes for degradation (Deng et al., 2016). In this context, it is worth noting that DISC1 interacts with APP and BACE1 (Deng et al., 2016; Shahani et al., 2015; Young‐Pearse, Suth, Luth, Sawa, & Selkoe, 2010). Recent studies observe that DISC1 distributes in both the outer and inner membrane of mitochondria (Park et al., 2010; Piñero‐Martos et al., 2016) and is required in maintenance of mitochondrial homeostasis such as ATP production and calcium buffering (Norkett, Modi, & Kittler, 2017; Park et al., 2010; Piñero‐Martos et al., 2016). Disrupted‐in‐schizophrenia‐1 also regulates axonal trafficking of mitochondria (Norkett et al., 2017). These lines of evidence suggest that DISC1 may be an essential factor in maintenance of mitochondrial homeostasis. We herein observe DISC1 binds directly to LC3‐I/II via a canonical LIR motif (^210^FSFI^213^). Overexpression of DISC1 induces mitophagy, which is dependent on its binding to LC3. Whereas knocking‐down of DISC1 blocks mitophagy induced by either Aβ or CCCP, both of which decrease mitochondrial membrane potential (Manczak et al., 2010; Ye, Sun, Starovoytov, & Cai, 2015). We further show that overexpression of DISC1 rescues Aβ‐induced mitochondrial dysfunction, loss of spines, and suppressed synaptic plasticity, in a manner dependent on its binding to LC3. Moreover, overexpression of DISC1, but not mutant DISC1, which harbors a mutated LIR motif and fails to induce mitophagy, via AAV8 in the hippocampus of 8‐month‐old APP/PS1 transgenic mice, rescues mitochondrial dysfunction and cognitive deficits, which is concomitant with attenuated synaptic loss and Aβ accumulation in the brains of these transgenic mice. Therefore, these results indicate that DISC1 protects synaptic plasticity from Aβ‐induced toxicity through acting as a mitophagy receptor.

## 2 RESULTS

### 2.1 DISC1 expression is downregulated in the brains of AD patients, which is associated with Aβ accumulation

**Figure 1:**
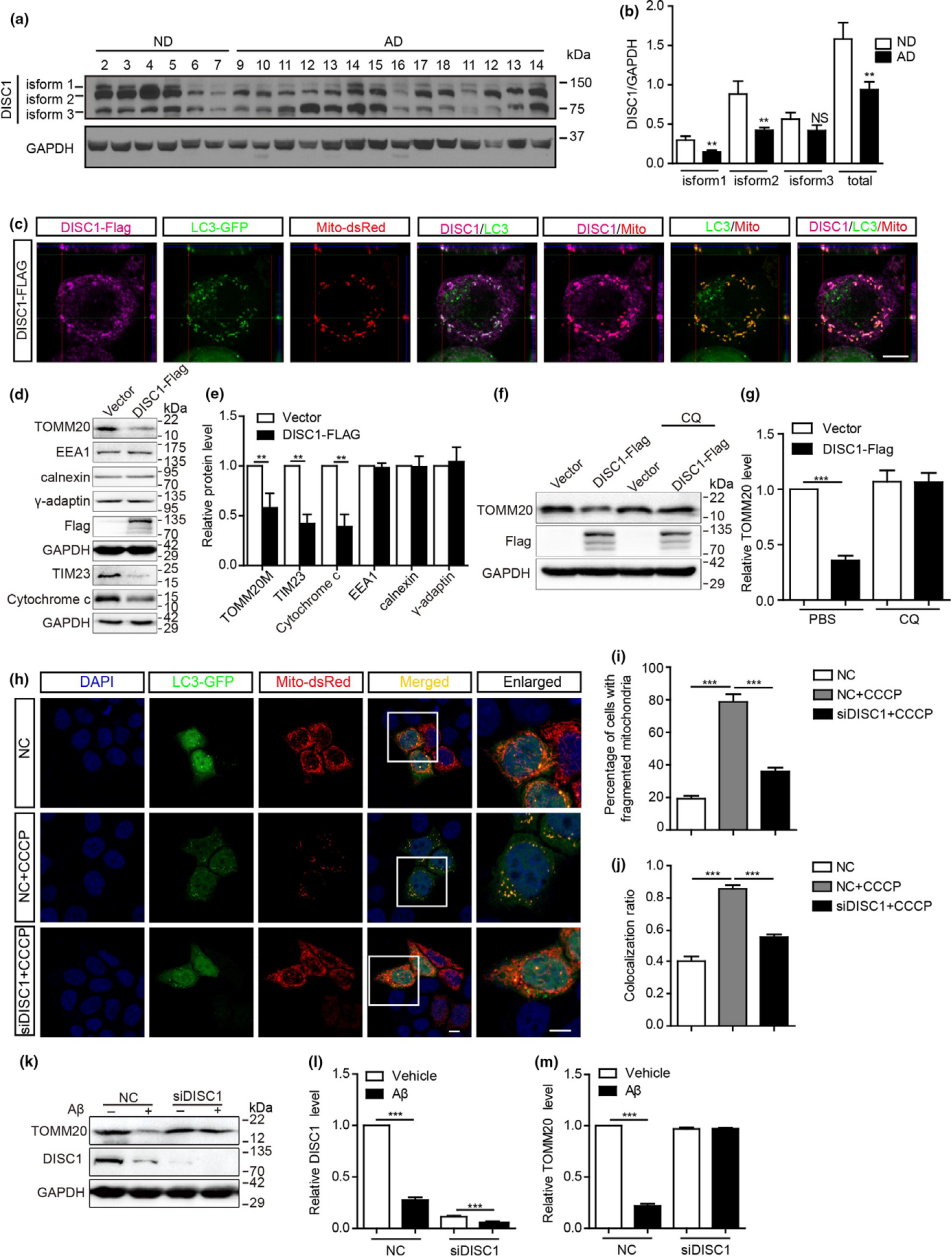
Downregulation of Disrupted‐in‐schizophrenia‐1 (DISC1) in the brains of AD patients and DISC1 is required in CCCP/Aβ‐induced mitophagy. (a) Western blot analysis of levels of DISC1 in the prefrontal cortex of AD patients. ND: age‐matched non‐AD patients. (b) Quantification of the levels of distinct DISC1 isoforms. (C) HeLa cells co‐transfected with mito‐dsRed, LC3‐GFP, and DISC1‐Flag were stained for Flag and imaged. Z‐stack image was shown. Scale bar: 10 μm. (d) Western blot analysis of levels of TOMM20, TIM23, cytochrome c, EEA1, calnexin, and γ‐adaptin in HeLa cells transfected with DISC‐Flag. (e) Relative levels of the above proteins were analyzed. (f) HeLa cells transfected with DISC1‐Flag or vector were treated with 20 μM chloroquine (CQ). Western blot analysis of levels of TOMM20. (g) Relative levels of TOMM20 were analyzed. (h) HeLa cells co‐transfected with LC3‐GFP, mito‐dsRed, and DISC1 siRNA or its scrambled siRNA (NC) were treated with 5 μM CCCP or DMSO for 6 hr and imaged. (i) Percentages of cells containing fragmented mitochondria. (j) Colocalization ratio between LC3‐GFP and mito‐dsRed. (k) HeLa cells were transfected with DISC1 siRNA or its scrambled siRNA (NC) and treated with 1 μM Aβ42. Levels of DISC1 and TOMM20 were analyzed with western blotting. (l, m) Quantification of relative levels of DISC1 (k) and TOMM20 (l). Data are presented as mean + *SEM*. *n* = 6 (ND), 14 (AD) (a, b). *n* = 3 or 4 independent experiments (c–m). **p < 0.01; ***p < 0.001. Two‐tailed independent *t* test (b, e) or one‐way ANOVA (g–m). Scale bars: 10 μm

To further identify the link between DISC1 and AD pathogenesis, we analyzed the levels of DISC1 in the prefrontal cortex of postmortem AD patients who were diagnosed by neurologists. The gender and age of these patients are listed in Supporting information Figure S1A. Disrupted‐in‐schizophrenia‐1 has multiple isoforms due to alternative splicing. At the protein level, the majority of DISC1 isoforms are detected between 100 and 130 kDa. A smaller isoform of DISC1 is also observed at 70–85 kDa, which reflects a form of DISC1 containing the amino acids in exons 2 and 5 (Ishizuka, Paek, Kamiya, & Sawa, 2006). We observed decreased levels of DISC1 isoforms at 100–130 kDa, rather than the smaller DISC1 isoform (75 kDa), in the cortex of AD patients compared with those in age‐matched people without AD (Figure 1a,b and Supporting information Figure S1). Consistently, DISC1 shows decreased levels in the brains of 8‐month‐old APP/PS1 transgenic mice, which exhibit AD‐like symptoms such as cognitive deficits, accumulation of Aβ, and loss of synapses. In contrast, DISC1 remains comparable levels to wild‐type mice in the brains 4‐month‐old APP/PS1 transgenic mice, which only harbor a few Aβ plaques (Deng et al., 2016). We thus wondered the downregulation of DISC1 in AD patients’ brain is linked to Aβ accumulation. As expected, DISC1 protein (Figure 1k), but not DISC1 mRNA (Supporting information Figure S2B), exhibited decreased levels upon being treated with Aβ oligomers in HeLa cells. These results indicate that DISC1 is downregulated in AD brains, which is caused by Aβ accumulation, suggesting a close link between DISC1 and AD pathogenesis.

### 2.2 DISC1 is required in Aβ‐/CCCP‐induced mitophagy

Disrupted‐in‐schizophrenia‐1 is a multi‐compartmentalized protein, which localizes at both the inner mitochondrial membrane (IMM) and the outer mitochondrial membrane (OMM) (Park et al., 2010; Piñero‐Martos et al., 2016). We observed parts of DISC1 distribute at the mitophagosomes as revealed by a colocalization between DISC1^+^ and GFP^+^RFP^+^ puncta in HeLa cells, which were co‐transfected with DISC1‐Flag, LC3‐GFP, and Mito‐dsRed plasmids (Figure 1c). We further observed transfection of DISC1‐Flag decreased levels of TOMM20 (a mitochondrial outer membrane protein), TIM23 (the major protein translocase of the mitochondrial inner membrane), and cytochrome c (an essential component of the electron transport chain) in HeLa cells. In contrast, proteins expressed in other organelles, such as early endosome antigen 1 (EEA1, a marker for early endosome), calnexin (a marker for the endoplasmic reticulum), and γ‐adaptin (a marker for the trans‐Golgi network), remained unchanged by transfection of DISC‐Flag (Figure 1d, e). In addition, the downregulation of TOMM20 by overexpression of DISC1 was prevented in the presence of chloroquine (CQ) (Figure 1f, g), which neutralizes the lysosomal pH (Klionsky et al., 2016). These results indicate that overexpression of DISC1 reduces levels of mitochondrial proteins via lysosomal degradation, suggesting a role of DISC1 in inducing mitophagy.

To further confirm this idea, we examined the role of endogenous DISC1 in mitophagy. HeLa cells were transfected with DISC1 siRNA, which could decrease the levels of DISC1 mRNA (Supporting information Figure S2A) and protein (Figure 1l) and treated with carbonyl cyanide 3‐chlorophenylhydrazone (CCCP), a respiratory chain uncoupler which reduces mitochondrial membrane potential and induces mitophagy (Ye et al., 2015). As described (Vives‐Bauza et al., 2010), treatment with CCCP induced mitochondrial fragmentation and mitophagy in HeLa cells as indicated by more cells containing fragmented mitochondria (Figure 1h, i), which were colocalized with LC3‐GFP^+^ puncta (Figure 1h, j). In contrast, such effects of CCCP were diminished in DISC1 siRNA‐knocking‐down cells (Figure 1h‐j). Oligomeric Aβ induces mitophagy and suppressed mitochondrial membrane potential in cultured neurons (Manczak et al., 2010; Ye et al., 2015). Consistently, we observed Aβ oligomers decreased TOMM20 levels in HeLa cells, whereas they failed to do so in DISC1‐siRNA‐transfected HeLa cells (Figure 1k, m). These results indicate that endogenous DISC1 is required in Aβ‐induced mitophagy.

### 2.3 DISC1 binds directly to LC3 through LIR motif

**Figure 2:**
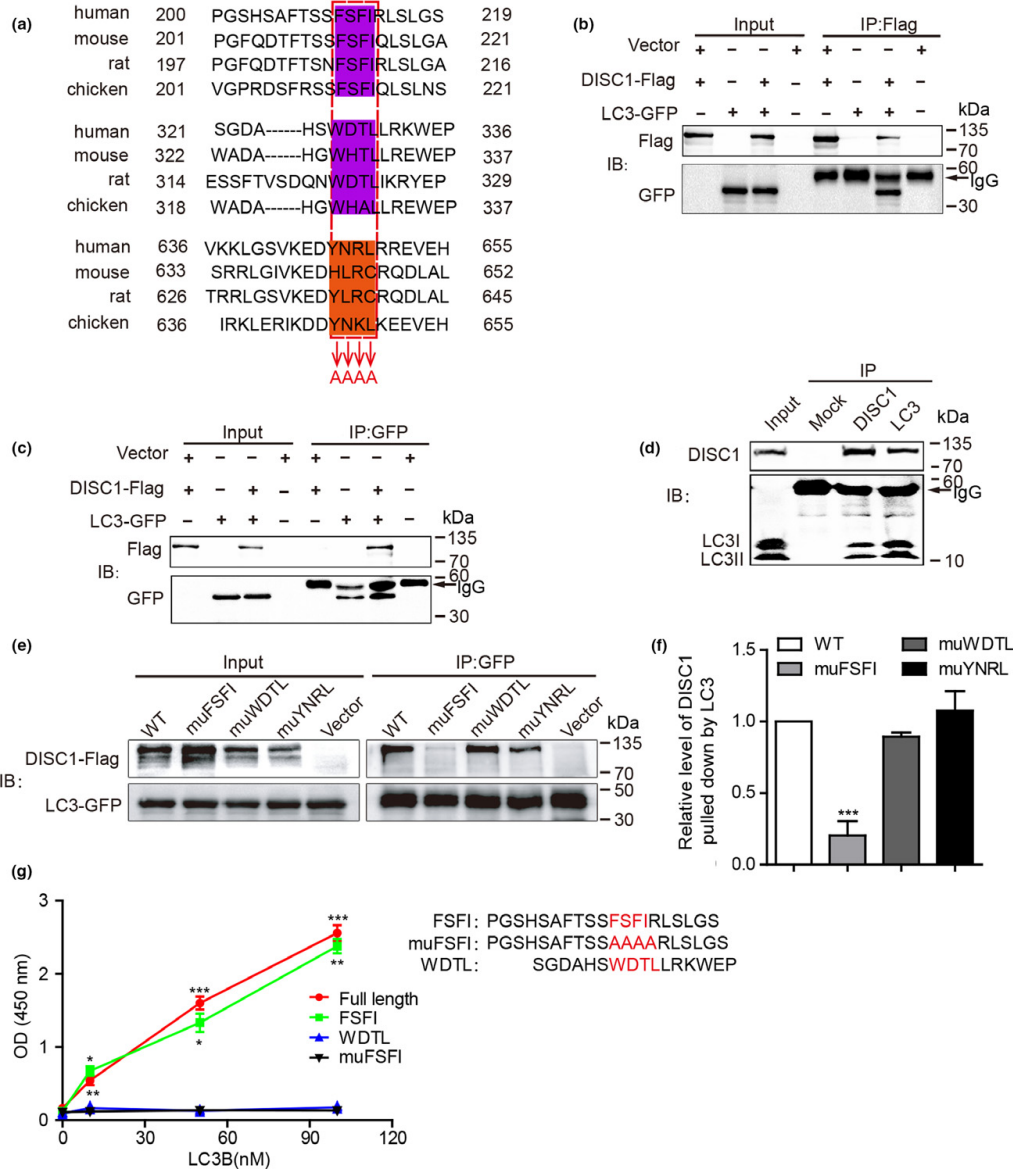
Disrupted‐in‐schizophrenia‐1 (DISC1) binds directly with LC3 via LIR motif. (a) Schematic illustration of LIR motifs in human DISC1. Conserved LIR motifs in DISC1 were highlighted in purple, which were mutated into AAAA in DISC1 LIR mutants. Amino acids highlighted in orange were also mutated into AAAA as control. (b, c) Lysates of HeLa cells co‐transfected with DISC1‐Flag and LC3‐GFP were immunoprecipitated with Flag (b) and GFP (c) antibodies, and probed with Flag and GFP antibodies, respectively. Cell lysates were loaded as input. (d) Lysates of 8‐month‐old APP/PS1 brain were immunoprecipitated with antibodies against DISC1 and LC3 and detected with antibodies against DISC1 and LC3, respectively. Non‐immune immunoglobulins (IgG) were used as control (Mock). (e) HeLa cells co‐transfected with LC3‐GFP and DISC1 WT‐Flag or DISC1 mutants‐Flag as indicated. Cell lysates were immunoprecipitated using immobilized anti‐GFP agarose and detected with anti‐GFP and anti‐Flag antibodies. Cell lysates were loaded as input. (f) The relative levels of DISC1 pulled down by LC3‐GFP were quantified. DISC1 levels pulled down by LC3‐GFP in DISC1 WT‐transfected cells were set to 1.0. (g) ELISA analysis of the binding of full‐length DISC1, peptides containing LIR motifs (FSFI) and its mutant (muFSFI), and control peptides (WDTL) to recombinant LC3 peptides which were coated on the wells. Binding was probed with anti‐LC3 antibody. Data are presented as mean + *SEM*. *n* = 3 or 4 independent experiments. *p < 0.05, **p < 0.01, and ***p < 0.001. One‐way ANOVA (e–g)

To identify the mechanism underlying DISC1‐mediated mitophagy, we examined in silico the DISC1 sequence and found that DISC1 possesses two potential LIR motifs: ^210^FSFI^213^ and ^327^WDTL^330^ (refer to human DISC1) (Figure 2a), suggesting an interaction between DISC1 and LC3. Co‐immunoprecipitation confirmed the binding between these two proteins using lysates of HeLa cells, which were co‐transfected with DISC1‐FLAG and LC3‐GFP. Antibodies against FLAG or GFP immunoprecipitated both DISC1‐Flag and LC3‐I/II‐GFP (Figure 2b,c). Moreover, co‐immunoprecipitation using brain lysates from 8‐month‐old APP/PS1 transgenic mice further confirmed an endogenous interaction between DISC1 and LC3‐I/II (Figure 2d). To further explore the specific LIR motifs on DISC1 interacting with LC3, we mutated these LIR motifs into quadruple alanine (Figure 2a) and performed immunoprecipitation analyses. ^646^YNRL^649^ which shares similar sequence with LIR motif was also mutated as control. The results showed that mutations in ^210^FSFI^213^ which is the most conserved LIR motif, but not in ^327^WDTL^330^ and ^646^YNRL^649^, nearly abolished the binding of DISC1 to LC3 (Figure 2e,f), indicating that DISC1 binds to LC3 via ^210^FSFI^213^. We further examined whether a direct interaction between DISC1 and LC3 exists by an ELISA experiment. Recombinant LC3 protein was able to bind to synthetic peptides encoding full‐length DISC1 and short sequence containing FSFI (a.a.200–219), which were coated as the substrate. In contrast, LC3 failed to bind to peptides encoding amino acids containing mutant FSFI (muFSFI) and amino acids containing WDTL (a.a.636–655) (Figure 2g). These results indicate that DISC1 binds directly to LC3 through ^210^FSFI^213^. Mutations in ^210^FSFI^213^ abolish the binding of DISC1 to LC3.

### 2.4 DISC1 induces mitophagy dependent on its binding to LC3

**Figure 3:**
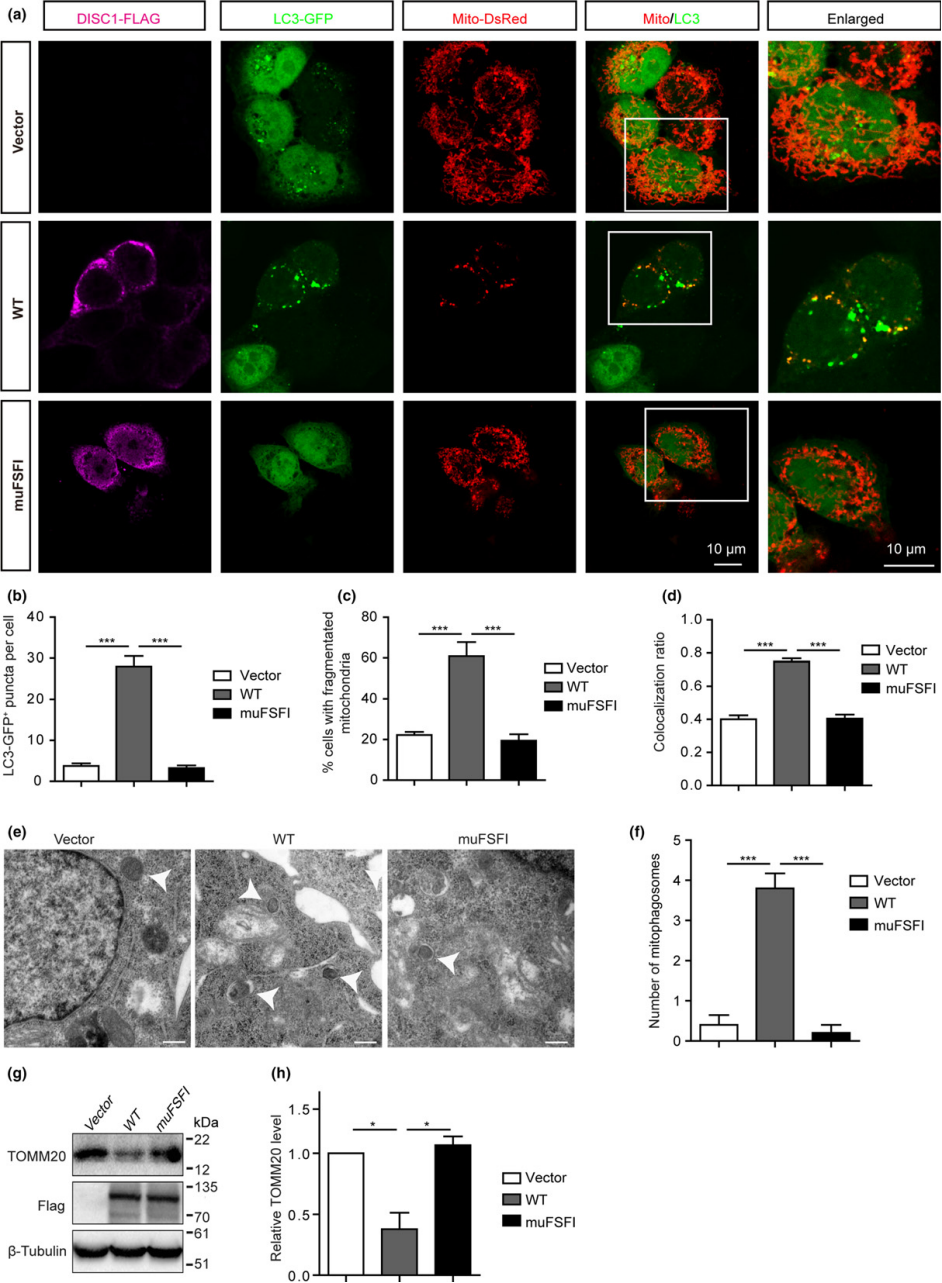
Disrupted‐in‐schizophrenia‐1 (DISC1) promotes mitophagy in a LIR‐dependent manner. (a) HeLa cells transfected with LC3‐GFP, mito‐dsRed, and DISC1‐Flag or DISC1 FSFI mutant‐Flag were stained for Flag and imaged. (b) The densities of GFP‐LC3^+^ puncta were counted and expressed as numbers of GFP‐LC3^+^ puncta per cell. (c) Percentages of cells with fragmented mitochondria. (d) Colocalization ratio between LC3‐GFP^+^ and mito‐dsRed^+^ puncta. (e) Mitophagosomes in HeLa cells transfected with DISC1 or DISC1 FSFI mutant were imaged by EM. (f) Numbers of mitophagosomes per cell. (g) Western blot analysis of levels of TOMM20 in HeLa cells transfected with DISC1‐Flag (WT) or DISC1 FSFI mutant‐Flag. (h) Quantification of relative levels of TOMM20. Data are presented as mean ± *SEM*. *n* = 3 or 4 independent experiments. *p < 0.05; ***p < 0.001. One‐way ANOVA. Scale bars: 10 μm (a), 500 nm (e)

We further asked whether DISC1 promotes mitophagy dependent on its binding to LC3. HeLa cells co‐transfected with LC3‐GFP, mito‐dsRed, and wild‐type DISC1‐Flag (WT) or mutant DISC1‐Flag (muFSFI) which abolishes the binding of DISC1 to LC3 were stained for Flag and imaged. In comparison with empty vector‐transfected cells, WT DISC1‐transfected HeLa cells harbored more fragmented mitochondria as described previously (Millar, James, Christie, & Porteous, 2005) (Figure 3a,c), more LC3‐GFP^+^ puncta (Figure 3a, b) and increased numbers of mitophagosomes as indicated by enhanced colocalization between LC3‐GFP^+^ and Mito‐dsRed^+^ puncta (Figure 3a,d). Transmission electron microscopy observations also showed that the numbers of mitophagosomes were increased by overexpression of WT DISC1, rather than mutant DISC1 (muFSFI) (Figure 3e,f). Moreover, in contrast to WT DISC1, which decreased TOMM20 levels in HeLa cells upon transfection, transfection of mutant DISC1 (muFSFI) failed to alter TOMM20 levels (Figure 3g,h). Thus, these results indicate that DISC1 promotes mitophagy in a way dependent on its binding to LC3.

### 2.5 DISC1 attenuates Aβ‐induced mitochondrial dysfunction in a LC3‐binding‐dependent manner

**Figure 4:**
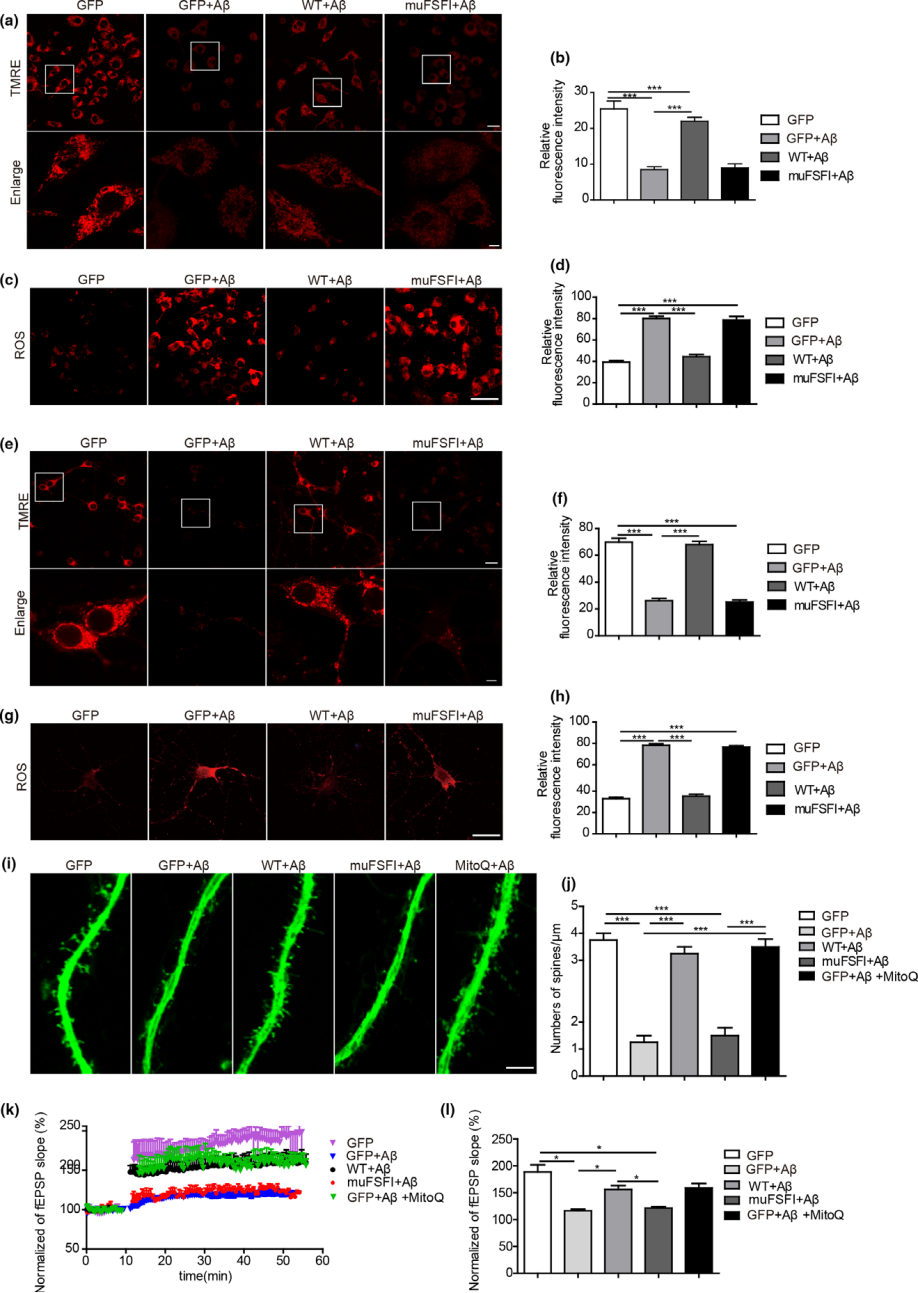
Disrupted‐in‐schizophrenia‐1 (DISC1) protects mitochondrial function from Aβ‐induced toxicity, which requires its binding to LC3. A‐H, SH‐SY5Y cells (a–d) or primary cortical neurons (e–h) infected with lentivirus carrying WT DISC1 (WT), DISC1 LIR mutant (muFSFI), or GFP were treated with 1 μM Aβ42 and loaded with TMRE (a, b, e, f) or analyzed levels of ROS (c, d, g, h). Relative fluorescent density was analyzed. (i, j) Primary cortical neurons infected with lentivirus carrying WT DISC1 (WT), DISC1 LIR mutant (muFSFI), or GFP were treated with 1 μM Aβ42 and either 20 nM MitoQ or DMSO (i). Densities of dendritic spines were quantified and expressed as numbers of spines per μm (j). (k, l) Acute hippocampal slices from C57/bl mice, which were injected hippocampally with lentivirus carrying WT DISC1 (WT), DISC1 LIR mutant (muFSFI), or GFP, were treated with 500 nM Aβ42 and 500 nM Mito Q or DMSO. LTP was recorded. The effects of high‐frequency‐stimulation (HFS) on the fEPSP initial slope (k). Cumulative data showing the mean fEPSP peak slope 60 min post‐HFS (l). Data are presented as mean + *SEM*. *n* = 4 or 5 independent experiments (a–j). *n* = 4 or 5 slices (l). *p < 0.05; ***p < 0.001. One‐way ANOVA

Parts of Aβ localize to mitochondria where they can cause mitochondrial dysfunction as evidenced by increased production of ROS and depolarization of membrane potential (Δψ_m_) (Manczak et al., 2010). To assess whether DISC1‐mediated mitophagy is associated with Aβ‐induced mitochondrial damage, DISC1 and its mutant form, muFSFI, were lentivirally transfected to SH‐SY5Y cells which were treated with 1 μM Aβ42 oligomers for 24 hr. The Δψ_m_ of mitochondria was assessed by the potential‐dependent fluorescent dye TMRE. Consistent with the previous report (Manczak et al., 2010), Aβ42 treatment induced depolarized Δψ as indicated by decreased fluorescent intensity for TMRE. In contrast, WT DISC1‐, but not muFSFI‐, transfected SH‐SY5Y cells failed to show depolarized Δψ upon Aβ42 treatment (Figure 4a,b). As described previously (Manczak et al., 2010), Aβ42 treatment increased ROS production as indicated by increased fluorescent density of an redox‐sensitive dye (CM‐H2DCFDA), that was rescued by overexpression of WT DISC1, rather than of muFSFI (Figure 4c,d). When the above experiments were performed in cultured primary cortical neurons, similar results were observed which show that WT DISC1, rather than mutant DISC1 (muFSFI), protects neurons from Aβ42‐induced mitochondrial damage (Figure 4e,f) and oxidative stress (Figure 4g,h). Thus, these results indicate that DISC1 protects cells from Aβ42‐induced mitochondrial dysfunction through promoting mitophagy.

### 2.6 DISC1 protects synaptic plasticity against Aβ‐induced toxicity through mitophagy

Accumulation of Aβ oligomers causes loss of dendritic spines and suppression of synaptic plasticity (Walsh et al., 2002; Wei et al., 2010). We then ask whether DISC1‐mediated mitophagy participates in the synaptic toxicity caused by Aβ accumulation. Since DISC1 levels decreased upon Aβ treatment (Figure 1k,l), we infected cortical neurons with lentivirus encoding WT DISC1 or muFSFI, which were cultured for 3 weeks and treated with Aβ42 oligomers. As reported previously (Shankar et al., 2007), treatment with Aβ42 oligomers decreased densities of dendritic spines. However, the loss of dendritic spines upon Aβ treatment was prevented when neurons were treated with MitoQ (mitoquinone mesylate: [10‐(4,5‐dimethoxy‐2‐methyl‐3,6‐dioxo‐1,4‐cycloheexadienl‐yl) decyl triphenylphosphonium methanesulfonate]), a mitochondria‐targeted antioxidant (Manczak et al., 2010) (Figure 4i,j), indicating that oxidative stress caused by mitochondrial dysfunction is involved in Aβ‐induced spine loss. Moreover, WT DISC1‐, but not muFSFI‐, transfected neurons also failed to show decreased densities of spines upon Aβ treatment (Figure 4i,j), indicating that transfection of DISC1 rescues Aβ accumulation‐induced spinal loss through enhancing mitophagy.

To examine whether DISC1‐mediated mitophagy is involved in Aβ‐suppressed synaptic plasticity, WT DISC1 or muFSFI (Supporting information Figure S2C,D) were lentivirally bilaterally injected into the hippocampus of 2‐month‐old C57BL/6 mice. Two months after injection, the hippocampal slices were treated with 500 nM Aβ42 oligomers and recorded long‐term potentiation (LTP). Treatment with Aβ42 oligomers induced suppressed LTP, which was prevented by cotreatment with MitoQ (Figure 4k,l), indicating mitochondrial dysfunction contributes to Aβ‐induced suppressed synaptic plasticity. Moreover, Aβ42 oligomers failed to decrease LTP in the WT DISC1‐transfected hippocampal slices. In contrast, Aβ42 oligomers still exhibited the capability in suppressing LTP in the muFSFI‐transfected hippocampal slices (Figure 4k,l and Supporting information Figure S2C,D). These results indicate that transfection of DISC1 rescues Aβ‐suppressed synaptic plasticity in a way dependent on DISC1‐mediated mitophagy. In summary, these results indicate that DISC1 rescues Aβ‐induced mitochondrial damage, oxidative stress, spine loss, and LTP impairments through enhancing mitophagy.

### 2.7 Overexpression of DISC1 rescues cognitive deficits of APP/PS1 transgenic mice

**Figure 5:**
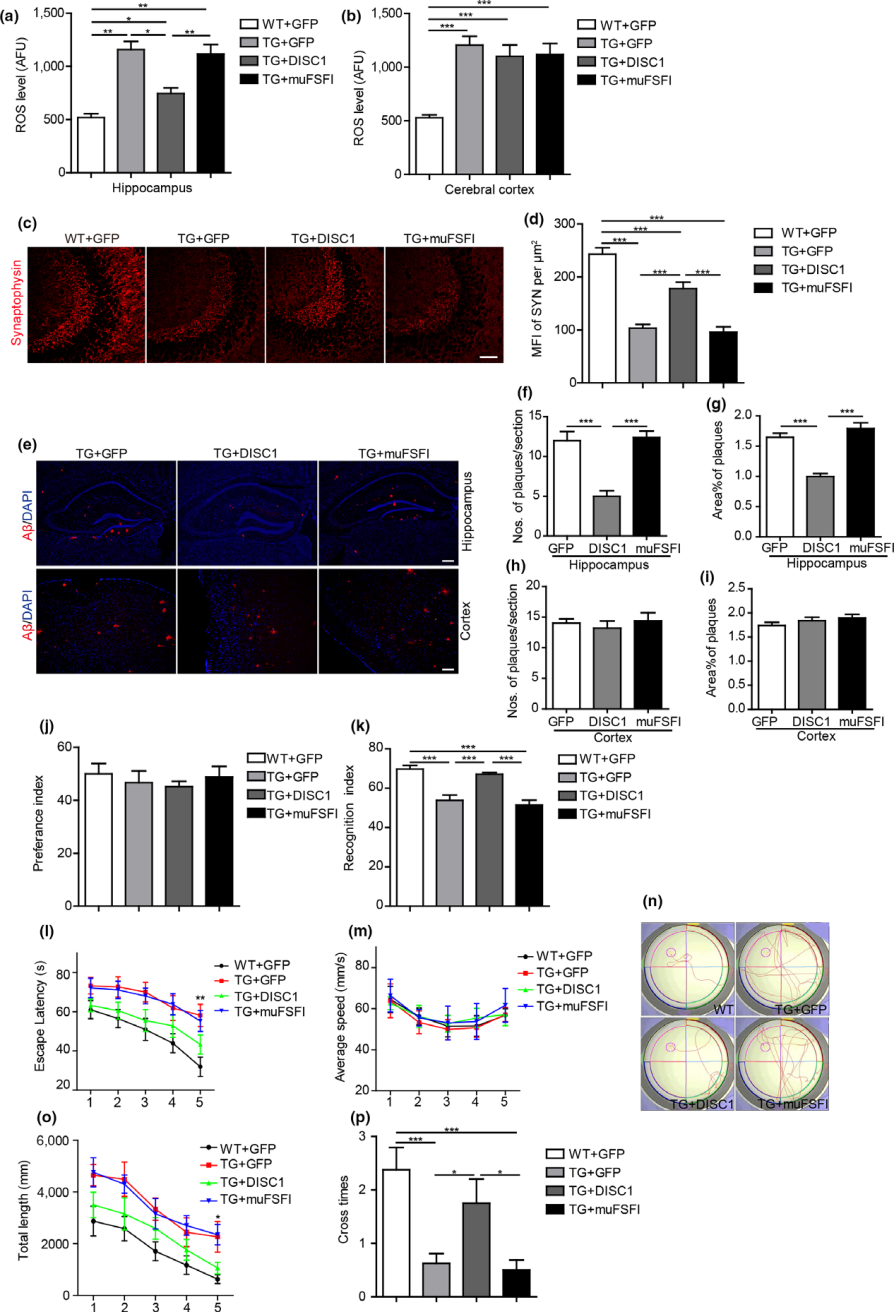
Overexpression of Disrupted‐in‐schizophrenia‐1 (DISC1) rescues cognitive deficits of APP/PS1 transgenic mice in an LC3‐depdendent manner. 8‐month‐old APP/PS1 transgenic mice were injected with AAV8 encoding DISC1‐Flag, muFSFI or GFP and subjected to behavioral analysis 4 months later. (a, b) Analysis of ROS levels in the hippocampus (a) and cortex (b) of the mice using ELISA. (c, d) The coronal sections of the hippocampal CA3 region were immunostained for synaptophysin (c). Mean fluorescent immunoreactivity (MFI) of synaptophysin was shown (d). (e–i) The coronal sections of the hippocampus and cortex were immunostained for Aβ and DAPI. Numbers (f, h) and size (g, i) of Aβ plaques in the hippocampus and cortex were quantified. (j, k) Novel object recognition test. Preference index (j), recognition index (k). L‐P, Morris water maze test. Escape latencies (l), average swimming speeds (m), representative images of swimming paths (n), distances swum (o), number of platform site crossings in the probe trials (p). Data are presented as mean + *SEM*. *n* = 8 mice/group. *p < 0.05, **p < 0.01, and ***p < 0.001. One‐way ANOVA (a–k, p) or two‐way ANOVA (l–o). Scale bars: 50 (c), 200 (e) μm

To examine whether overexpression of DISC1 ameliorates cognitive deficits through enhancing mitophagy, we injected bilaterally AAV8 encoding either DISC1, muFSFI, or GFP into the hippocampus of 8‐month‐old APP/PS1 transgenic mice and performed behavioral tests 4 months after injection. APP/PS1 transgenic mice exhibit the appearance of amyloid plaques at 3‐months old of age and extensive accumulation of amyloid plaques in the brains at 8‐months old of age (Zhang et al., 2014). Disrupted‐in‐schizophrenia‐1 reduces Aβ generation through promoting lysosomal degradation of BACE1. Overexpression of DISC1 at 4‐months of age attenuates accumulation of amyloid plaques, and thus cognitive deficits in these transgenic mice (Deng et al., 2016). Thus, to examine the effects of DISC1‐mediated mitophagy on cognitive deficits, we injected AAV8 encoding DISC1 in the hippocampus of APP/PS1 transgenic mice at 8 months old of age, when these mice already harbored extensive accumulation of amyloid plaques in the brains. Moreover, most of AD patients were diagnosed at relative late stage when their brains already contain extensive Aβ load (He et al., 2017; Musiek & Holtzman, 2015). Overexpression of DISC1 at 8‐month‐old transgenic mice could allow us to examine the therapeutic effects of DISC1 on AD pathogenesis. Thus, we injected bilaterally AAV8 encoding DISC1 or its mutant form in the hippocampus of 8‐month‐old APP/PS1 transgenic mice. The results revealed that overexpression of WT DISC1 hippocampally, rather than muFSFI, rescued increased ROS levels in the hippocampus (Figure 5a) of APP/PS1 transgenic mice, indicating that DISC1 rescues mitochondrial dysfunction in these AD model transgenic mice through enhancing mitophagy. In contrast, ROS levels in the cortex, where no AAV8‐DISC1 was injected, remained unchanged among these groups of mice (Figure 5b). In addition, overexpression of WT DISC1 hippocampally attenuated loss of synapses as evidenced by enhanced synaptophysin^+^ immunoreactivity, a marker for synapses, in the hippocampus of APP/PS1 transgenic mice (Figure 5c,d). Overexpression of DISC1 reduced the densities of Aβ (the numbers and the size of amyloid plaques) in the hippocampus (Figure 5e–g), but not in the cortex (Figure 5e,h,i), of APP/PS1 transgenic mice. In contrast, overexpression of muFSFI, which abolishes DISC1‐mediated mitophagy, failed to alter synaptophysin^+^ immunoreactivity and Aβ plaques (Figure 5c–i).

Behavioral tests were performed to examine cognition of these transgenic mice. In novel object recognition test, no significant difference was observed in preference index during the training phase among the groups, indicating that the objects do not affect the exploratory behavior of mice (Figure 5j). In the testing phase, GFP‐injected transgenic mice show a decreased recognition index (RI) than GFP‐injected WT mice (Figure 5k), indicating cognitive deficits. However, injection of AAV8 encoding DISC1(WT), but not muFSFI, improved RI in APP/PS1 mice, even to a level comparable to that in WT mice (Figure 5k), indicating that overexpression of DISC1 hippocampally rescues cognitive deficits of APP/PS1 transgenic mice. In Morris water maze test, GFP‐injected transgenic mice were impaired in learning to use the available visuospatial cues to locate the submerged platform, as indicated by their longer escape latency (Figure 5l) and increased swimming distances (Figure 5n,o) compared to AAV8‐GFP‐injected wild‐type mice. In contrast, DISC1‐injected transgenic mice exhibited shorter escape latency (Figure 5l) and swimming distances (Figure 5n,o) compared to GFP‐injected transgenic mice, and even approached a level comparable to GFP‐injected WT mice. The changes in escape latency were not due to the differences in swimming speed, which were identical among the groups (Figure 5m). In the probe trial, DISC1‐injected transgenic mice showed improved memory retention as indicated by that they swam to cross over the target site more times than GFP‐injected APP/PS1 mice (Figure 5p). However, muFSFI‐injected mice exhibited no differences compared to GFP‐injected transgenic mice in the above behavioral tests (Figure 5l–p). These results indicate that overexpression of DISC1 hippocampally rescues cognitive deficits of APP/PS1 mice through promoting mitophagy.

## 3 DISCUSSION

### 3.1 DISC1 is a novel mitophagy receptor

**Figure 6:**
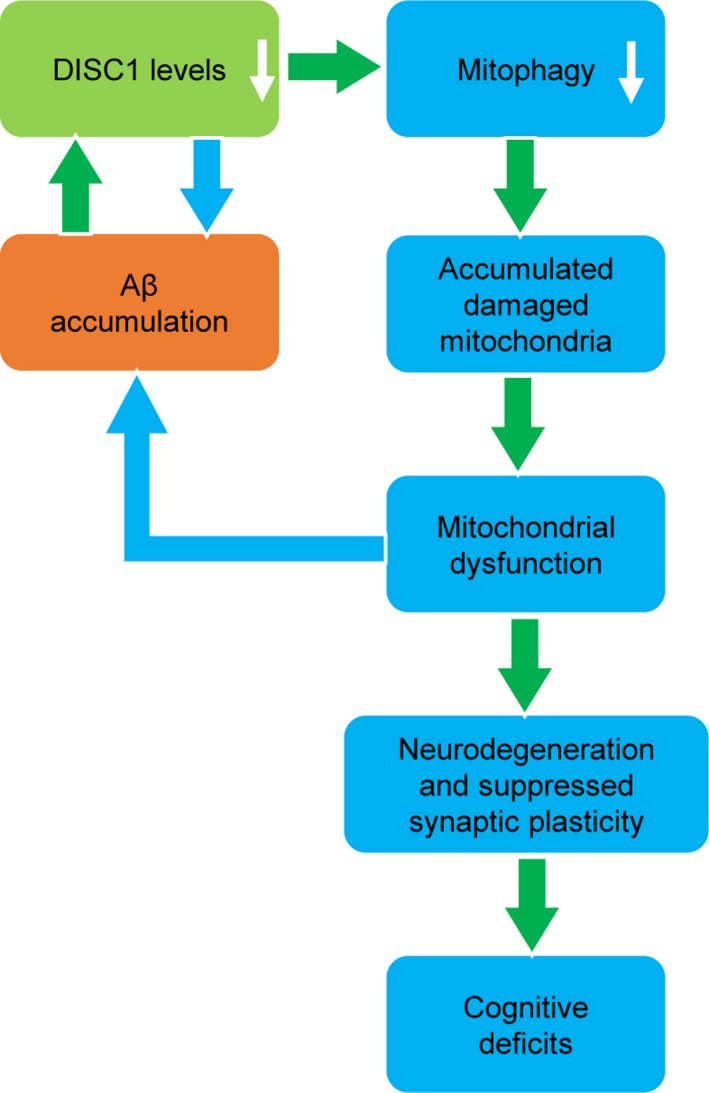
Schematic description of Disrupted‐in‐schizophrenia‐1 (DISC1)‐mediated mitophagy in AD pathogenesis. DISC1 is a mitophagy receptor, which binds directly to LC3. DISC1 levels are downregulated in response to Aβ accumulation. Downregulation of DISC1 results in impaired mitophagy, thus accumulation of damaged mitochondria. Mitochondrial dysfunction causes neurodegeneration and suppressed synaptic plasticity. In other hand, mitochondrial dysfunction exacerbates Aβ accumulation. In this way, DISC1 levels and Aβ accumulation form a vicious cycle, which eventually leads to cognitive deficits in AD

Multiple mitophagy receptors have been identified, such as p62, NBR1, Nix, Tax1BP1, NDP51, optineurin, and FUNDC1, that direct the assembling autophagosomes to surround the damaged mitochondria through binding to LC3 (Lazarou et al., 2015; Liu et al., 2012). We herein identify that DISC1 is a novel mitophagy receptor, which binds to LC3 via a canonical LIR motif. We further show that DISC1 is required in Aβ‐mediated mitophagy and protects synaptic plasticity against Aβ‐induced toxicity as a mitophagy receptor. In the brains of AD patients and transgenic mice, DISC1 levels are downregulated by accumulated Aβ. The downregulation of DISC1 results in mitochondrial dysfunction via impairing mitophagy, that causes suppressed synaptic plasticity, eventually leading to deficits in learning and memory (Figure 6). Since mitochondrial dysfunction further exacerbates Aβ generation (Kerr et al., 2017), DISC1 levels and Aβ generation thus form a vicious cycle, which together promotes AD pathogenesis. Disrupted‐in‐schizophrenia‐1 is a novel therapeutic target for AD.

Either Aβ or CCCP causes depolarization of mitochondrial membrane potential, thus inducing mitophagy (Vives‐Bauza et al., 2010; Ye et al., 2015). Knockdown of DISC1 prevents Aβ‐ or CCCP‐induced mitophagy, indicating that DISC1 is required in Aβ‐ or CCCP‐induced mitophagy. It is worth noting that in the absence of Aβ, knockdown of DISC1 fails to decrease TOM20 levels (Figure 1k,m). These results indicate that DISC1‐mediated mitophagy is triggered by mitochondrial damage. Parkin is translocated to the damaged mitochondrial following the treatment with CCCP or Aβ (Vives‐Bauza et al., 2010; Ye et al., 2015). Following translocation to the mitochondrial surface, Parkin ubiquitylates and degrades numerous OMM proteins such as mitofusin (MFN) 1 and 2, voltage‐dependent anion channel, mitochondrial Rho GTPases (MIRO) 1 and 2, and some components of the translocase of the OMM (TOM) complex (TOM70, TOM40, and TOM20), which in turn recruit other proteins to mitochondria to initiate mitophagy (Jin & Youle, 2012). Among them, DISC1 forms an interacting complex with MIRO1, MFN1 (Norkett et al., 2017). However, overexpression of DISC1 induces robust mitophagy in HeLa cells, where few Parkin is expressed (Strappazzon et al., 2015), suggesting that DISC1 promotes mitophagy in a Parkin‐independent way. In addition, consistent with the fact that the OMM is the site where mitophagy is initiated, mitophagy receptors mentioned above are expressed at the OMM (Lazarou et al., 2015; Liu et al., 2012). However, a recent finding indicates that IMM proteins such as prohibitin2 (PHB2), an IMM protein, also serve as a mitophagy receptor. PHB2 binds to LC3 only after OMM is ruptured, which occurs in Parkin‐dependent mitophagy (Y. Wei, Chiang, Sumpter, Mishra, & Levine, 2017). In this context, it is worth noting that DISC1 localizes at both the OMM and IMM (Park et al., 2010; Piñero‐Martos et al., 2016). Therefore, it remains to be further investigated whether and how DISC1 in the OMM or/and IMM mediates mitophagy.

Our previous study indicates that DISC1 exhibits decreased levels in the brains of APP/PS1 transgenic mice only at 8‐months old of age when extensive Aβ plaques are accumulated (Deng et al., 2016). Consistent with this finding, we herein observe that DISC1 levels are decreased posttranscriptionally by Aβ treatment. Disrupted‐in‐schizophrenia‐1 levels are increased by the binding of neuregulin to ErbB2/3, which requires activation of PI3K/Akt signaling (Seshadri et al., 2010). Aβ interrupts PI3K/Akt signaling (Chen, Wang, & Chen, 2009), so it is possible that Aβ downregulates DISC1 levels through inhibiting PI3K/Akt. In addition, Aβ induces mitophagy (Ye et al., 2015). As a mitophagy receptor, DISC1 could also be degraded together with damaged mitochondria as mitophagy is induced. Despite the unclear mechanism underlying how Aβ treatment downregulates DISC1 expression, it seems a vicious feedback cycle existing between DISC1‐mediated mitophagy and Aβ accumulation. Aβ accumulation triggers DISC1 to induce mitophagy, which decreases DISC1 expression. Downregulation of DISC1 attenuates mitophagy, which in turn execrates mitochondrial dysfunction and toxicity induced by Aβ accumulation.

### 3.2 DISC1 protects synaptic plasticity from Aβ‐induced toxicity as a mitophagy receptor

Disrupted‐in‐schizophrenia‐1 is required in maintaining mitochondrial homeostasis. Knocking‐down of DISC1 results in reduced ATP production, decreased NADH dehydrogenase activity, slower calcium buffering, and impaired mitochondrial trafficking (Norkett et al., 2017; Park et al., 2010; Piñero‐Martos et al., 2016). We here report a novel role of DISC1 in maintaining mitochondrial homeostasis that DISC1 promotes mitophagy as a mitophagy receptor. Overexpression of DISC1 rescues Aβ‐induced mitochondrial dysfunction and impaired synaptic plasticity. In contrast, LIR mutant DISC1 (muFSFI), which still localizes at the mitochondria and abolishes the interaction of DISC1 with LC3 (Figure 4a), fails to do so. These results indicate that despite various functions of DISC1 in maintenance of mitochondrial health, its interaction with LC3 is required in protecting cells against Aβ‐induced toxicity, enlightening a contribution of mitophagy.

Disrupted‐in‐schizophrenia‐1 promotes lysosomal degradation of BACE1. Prophylactic expression of DISC1 in the hippocampus of 4‐month‐old APP/PS1 transgenic mice, when extensive Aβ plaques have not formed yet and the cognition is normal, ameliorates Aβ accumulation and cognitive deficits (Deng et al., 2016). To evaluate the effects of DISC1‐mediated mitophagy in cognition, we therapeutically express DISC1 in the hippocampus of APP/PS1 transgenic mice at 8 months old of age, when these mice harbor extensive Aβ plaques and loss of synapses, exhibiting cognitive deficits. Even though DISC1 is overexpressed at the relative late pathological stage, overexpression of DISC1 rescues dysfunctional mitochondria, synaptic loss, and defective cognition of these transgenic mice. Like prophylactic expression of DISC1, therapeutic expression of DISC1 also decreases accumulation of Aβ plaques. Since mitochondrial dysfunction initiates Aβ production, which in turn, when accumulates into a certain amount, exacerbates mitochondrial damage (Kerr et al., 2017), the decreases in Aβ plaque density may be a result from coordinate work by DISC1‐mediated mitophagy and BACE1 degradation. However, overexpression of DISC1 prevents loss of spines in cultured neurons and suppression of synaptic plasticity in hippocampal slices, which are induced by Aβ treatment. These results indicate that DISC1‐mediated mitophagy indeed preserves synaptic plasticity from Aβ‐induced toxicity, which is independent on its effect on Aβ generation. Impaired mitophagy caused by downregulation of DISC1 is a causal factor contributing to neurodegeneration in AD brains.

## 4 EXPERIMENTAL PROCEDURES

### 4.1 Animals

Male APP/PS1 transgenic mice that co‐express mutant human *APP* and *PS1* (Jackson Laboratory, 004462), which were maintained on a C57BL/6J background, were housed in specific pathogen‐free (SPF) conditions. Animal care and surgical procedures were approved by the Animal Studies Committee of Southern Medical University and of the Beijing Military Hospital in accordance with international laws.

### 4.2 Postmortem brain samples of human patients

Brain samples from AD patients and age‐matched non‐AD patients were from brain bank of Case Western Reserve University (Cleveland, USA). All patients were diagnosed by neurologists. Postmortem brain samples were collected 3–21 hr after death.

### 4.3 ELISA for analysis of proteins interaction

Human full‐length DISC1 protein and its truncated peptides including WDTL (SGDAHSWDTLLRKWEP), FSFI (PGSHSAFTSSFSFIRLSLGS), and its mutated forms, muFSFI (PGSHSAFTSSAAAARLSLGS), were synthesized by China peptides Company (Shanghai, China). Purified recombinant LC3 protein was purchased from Origene (Rockville, MD). 500‐nM full‐length DISC1 protein or the above truncated peptides in PBS were coated as substrates on 96‐well plates at 4°C for overnight. The wells coated with PBS as substrate were used as the control. After washing and blocking with 4% BSA in PBS, different concentrations of recombinant LC3 protein (0, 10, 50, 100 nM) were incubated for 1 hr at 37°C. Wells were then washed and incubated with mouse anti‐LC3 antibody followed by incubation with HRP‐conjugated goat anti‐mouse IgG. OD values were read using the TMB system (Invitrogen).

### 4.4 Statistical analysis

The data are presented as the mean ± *SEM*. Simple comparisons between two groups were analyzed using independent *t* tests. Multiple comparisons between the groups were performed using one‐way or two‐way ANOVA followed by post hoc analysis with LSD or Dunnett's T3 test as appropriate on SPSS 20.0 software. *p* < 0.05 was considered statistically significant.

Other materials such as antibodies, plasmids, siRNAs, viruses, and experimental procedures including behavioral tests, co‐immunoprecipitation, LTP recording, analysis of spine density, measurement TMRE, ROS, and TEM were described in the Supporting information Appendix S1.

## CONFLICT OF INTEREST

The authors have no conflicting financial interests.

## AUTHOR CONTRIBUTIONS

Zhaotao Wang participated in molecular experiments, prepared figures, and edited manuscript; Meihong Lv participated in immunofluorescence experiments; Yan Zhang was involved in electron microscope experiments; Wenli Ji was involved in western blots; Lei Lei contributed to recording of long‐term potentiation; Wang Wang contributed to synthetic plasmids; Lipao Fang participated in confocal macroscope image acquiring; Luwen Wang,Fan Yu, and Zhenyu Li participated in real‐time PCR; Fei Dou, Qinwen Wang, Jianrong Wang, Ting‐hua Wang, and Xinlong Wang were involved in western blots and quantification; Shao Li participated in LTP and planned experiments; Ruxiang Xu planned experiments; Quanhong Ma designed, planned experiments, and wrote manuscript.
