# Supplementary material for: Disrupted‐in‐schizophrenia‐1 protects synaptic plasticity in a transgenic mouse model of Alzheimer’s disease as a mitophagy receptor
Source: Aging Cell. 2018 Nov 28;18(1):e12860. doi: 10.1111/acel.12860 (PMC6351828; doi:10.1111/acel.12860)
Supplement: Supplementary file 1 [file ACEL-18-e12860-s001.pdf]

A

Samples of AD human brain lysates

Number	Diagnosis	Age	Gender
1	ND	75	Female
2	ND	80	Female
3	ND	53	Male
4	ND	65	Male
5	ND	74	Male
6	ND	77	Male
7	AD	64	Female
8	AD	76	Female
9	AD	82	Female
10	AD	82	Female
11	AD	83	Female
12	AD	89	Female
13	AD	93	Female
14	AD	60	Male
15	AD	72	Male
16	AD	80	Male
17	AD	57	Male
18	AD	74	Male
19	AD	80	Male
20	AD	80	Male

B

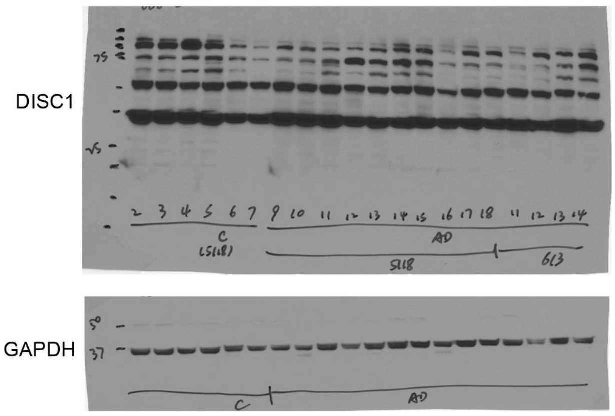

Supplementary fig. 1
