# Supplementary figures and images for: Disrupted‐in‐schizophrenia‐1 protects synaptic plasticity in a transgenic mouse model of Alzheimer’s disease as a mitophagy receptor
Source: Aging Cell. 2018 Nov 28;18(1):e12860. doi: 10.1111/acel.12860 (PMC6351828; doi:10.1111/acel.12860)

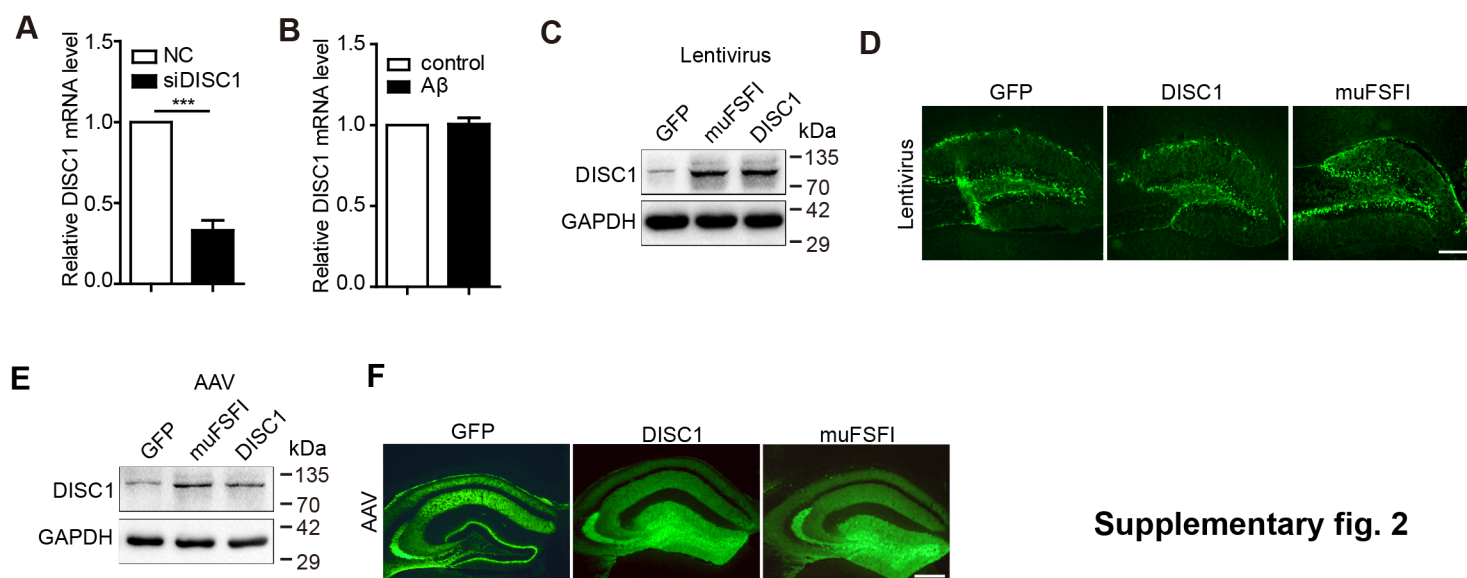

Supplementary fig. 2

Supplement: Supplementary file 2 [file ACEL-18-e12860-s002.pdf]
