# Supplementary material for: Disrupted‐in‐schizophrenia‐1 protects synaptic plasticity in a transgenic mouse model of Alzheimer’s disease as a mitophagy receptor
Source: Aging Cell. 2018 Nov 28;18(1):e12860. doi: 10.1111/acel.12860 (PMC6351828; doi:10.1111/acel.12860)
Supplement: Supplementary file 3 [file ACEL-18-e12860-s003.docx]

**Supplementary information**

**Supplementary figure legend**

**Supplementary figure 1. Detailed information about AD patients and the control patients.**

The gender and the age of the patients were shown. AD: Alzheimer’s disease patients; ND: non-AD patients.

**Supplementary figure 2.**

**A** and **B**, qPCR analysis of levels of DISC1 mRNA in HeLa cells which were transfected with DISC1 siRNA (**A**) or treated with 1 μM Aβ42 (**B**).

**C** and **D**, 2-momth-old C57/bl6 mice were injected hippocampally with lentivirus encoding GFP and WT DISC1 or muFSFI or GFP alone. DISC1 levels were analyzed 2 months after injection with western blotting using DISC1 antibody (**C**). The coronal sections of lentivirus-injected mice were imaged (**D**).

**E** and **F**, 8-month-old APP/PS1 transgenic mice were injected hippocampally with AAV8 encoding WT DISC1-FLAG, muFSFI-FLAG or GFP-FLAG. DISC1 levels were analyzed 2 months after injection with western blotting using DISC1 antibody (**E**). The coronal sections of AAV8 DISC1-, AAV8-muFSFI-injected mice were stained for FLAG (**F**). Scale bars: 200 μm.

**Materials and Methods**

**Antibodies.** Anti-DISC1 (#40-6800) are purchased from Thermo Fisher Scientific (Grand Island, New York); anti-LC3 (L8918), anti-Flag (F7425), anti-calnexin (C4731), anti-γ-adaptin (A4200) and anti-GFP (G1544) are obtained from Sigma-Aldrich (St. Louis, Missouri, USA); anti-EEA1 (#3288), anti-TOMM20 (#42406) and anti-GAPDH (#5174) were purchased from Cell Signaling Technology (Danvers, USA). Alexa Fluor-conjugated secondary antibodies were from Thermo Fisher Scientific; Horseradish peroxidase (HRP) -conjugated secondary antibodies were from Sigma-Aldrich.

**Plasmids, siRNAs and virus.** Human full-length DISC1 cDNA containing a C-terminus linked Flag was cloned into a pAOV vector. The pAOV DISC1-Flag-expression construct was used as a template to generate DISC1-Flag-muWDTL, DISC-Flag-muYNRL and DISC-Flag-muFSFI mutations. A pAOV vector-expressing GFP served as control. The LC3-GFP and Moti-dsRed plasmids are purchased from addgene. DISC1 siRNAs and its scrambled siRNA (NC) were from Genepharma (Shanghai, China). DISC1 siRNA (sense: GGCAGAUGGAUGACUUAGATT; anti-sense: UCUAAGUCAUCCAUCUGCCTT); NC siRNA (sense: UUCUCCGAACGUGUCACGUTT; anti-sense: ACGUGACACGUUCGGAGAATT). Lentivirus encoding GFP and either human full-length DISC1 or mutated DISC1 (muFSFI), AAV8 encoding human full-length DISC1 or mutated DISC1 (muFSFI), both of which are tagged by Flag were produced by GeneChem (Shanghai, China).

**Preparation of Aβ oliogomers.** 1.0 mg of synthetic Aβ42 was dissolved to 1 mM in hexafluorisopropanol (HFIP) for 60 min at room temperature. The Aβ42 solution was then separated into aliquots in microcentrifuge tubes and removed HFIP under vacuum in a Speed Vac (Sc110 Savant Instruments). The lyophilized peptide film was stored desiccated at -20 ℃. For experiments, the HFIP-free aliquots were resuspended in distilled water to a concentration of 50 mM. Then stock solutions were further diluted with PBS to 1 μM as the final concentration. Then it was incubated at 37 ℃ for 5 days to obtain oligomers.

**Cell culture and transfection.** HeLa cells and SH-SY5Y cells were cultured in Dulbecco’s modified Eagle’s medium (Invitrogen) supplemented with 10% fetal bovine serum (FBS), 2 mM L-glutamine. Cortical neurons were isolated from the telencephalon of embryonic day 16-18 C57BL/6 mice. Neurons were cultured in neurobasal medium containing 2% B27 supplement, 1% penicillin/streptomycin and 0.1% L-glutamine (Invitrogen) on poly-L-lysine-coated glass coverslips or plates. HeLa cells were transfected by Lipofectamin 3000 (Thermo Fisher Scientific) according to the manufacturers’ protocol. For transfection of SH-SY5Y cells and neurons, 2.5μL 2.8×10^11^ vector genome/ml lentivirus was added to the media.

**Analysis of densities of dendritic spines.** Neurons were infected with lentivirus encoding GFP and either WT DISC1 or muFSFI after being cultured for 14 days. The virus-infected neurons were cultured for a week and treated with 1 μM Aβ42 oligomers and either 20 nM MitoQ or DMSO for 12 h. The cells were then fixed with 4% paraformaldehyde and the images were acquired by laser confocal microscope. Then the images were imported into the software Image Pro-Plus and the number of spines in per unit length dendrite were counted.

**Reverse transcription polymerase chain reaction (RT-PCR).** Total cellular RNA was extracted using Trizol Reagent (Sigma-Aldrich). Equal amount of the first-strand cDNAs were synthesized with the FastQuant RT Kit (Tiangen Biotech, Beijing, China). PCR reactions were performed with Taq DNA polymerase (Takara, Dalian, China). The following primers were used. Mouse *DISC1*: GCACTTTGCGGTTCATTCCAA (Forward), GGAGCCAGAGACTTAAAGCTG (Reverse); Mouse *GAPDH*: TCCACCACCCTGTTGCTGTAG (Forward), GACCACAGTCCATGACATCACT (Reverse).

**Co-immunoprecipitation.** Mouse hippocampus and HeLa cells were lysed with RIPA buffer (50mM Tris buffer (pH 7.4), 150 mM NaCl, 1% Triton X-100, 1% sodium deoxycholate, 0.1% SDS) containing the protease inhibitor cocktail. The lysates were precleared with protein A/G conjugated agarose beads (Santa Cruz, Dallas) for 2 h and incubated together with antibodies and protein A/G conjugated agarose beads overnight at 4°C. The beads were washed 3 times with RIPA buffer, re-suspended in SDS electrophoresis sample buffer, and boiled for 5 min at 95°C. Samples were subjected to SDS-PAGE and Western blot analysis.

**Virus injection.** Lentivirus particles (2.8×10^11^ vector genome/ml) and AAV8 particles (5.4×10^12^ vector genome/ml) were injected bilaterally into the hippocampus (-2.1 mm anteroposterior from bregma, ±1.8 mm mediolateral from bregma and 1.8 mm below the surface of the skull) of 2-month-old C57BL/6 mice and 8-month-old APP/PS1 transgenic mice, respectively, by a stereotaxic apparatus. One µl of the virus suspension was injected into each hippocampus of a mouse through a 10 µl gauge needle at the rate of 0.2 µl/min. After injection, the needle was left in place for an additional 5 min before being slowly withdrawn.

**Recording of long-term potentiation (LTP)**. LTP was recorded in the hippocampus of C57BL/6 mice which were injected with lentivirus for 2 months. Acute transverse hippocampal slices (400 μm) were prepared from the resected brains of mice using a vibratome (Leica VT1200S). Field excitatory postsynaptic potential (fEPSP) was recorded in the molecular layer of the hippocampal DG region. Stimulating and recording electrodes were placed in the molecular layer of the dentate gyrus. The stimulation pulse (0.2 ms duration, 0.033 Hz) selected for baseline measurements was adjusted to yield 40% of its maximal slope. After baseline responses had stabilized for 30 mins, LTP was induced using high-frequency stimulation. The electrophysiological data were acquired with an Axon multiclamp 700B amplifier, filtered at 10×10^4^ kHz, and digitized at 10 kHz. The slope of fEPSP were measured and analyzed using pClamp10.3 software (Molecular Devices Corp, USA). For experiments on the dentate gyrus, all solutions contained 100 μM picrotoxin (Sigma, St. Louis, MO). The control medium contained (in mM): 120 NaCl, 2.5 KCl, 1.25 NaH_2_PO_4_, 26 NaHCO_3_, 1.3 MgSO_4_, 2.5 CaCl_2_, 10 d-glucose. 500 nM Aβ42 oligomers was perfused for 40 mins before high-frequency stimulation (HFS)-induced LTP. 500 nM MitoQ was added 60 mins before HFS.

**Immunofluorescence.** Cells were fixed in 4% paraformaldehyde for 15 min, and then were penetrated with PBS containing 0.01% Triton X-100 (PBST) for 10 min, followed by blocking with 10% BSA in PBS for 1 h, and incubated with primary antibody overnight at 4°C. Cells were washed 3 times in PBS and incubated with appropriate secondary Alexa fluor-conjugated antibodies for 2 h at room temperature. The sections were then washed 3 times with PBS and mounted in mounting medium containing DAPI (Vector Laboratories, Burlingame).

**Measurement of Mitochondrial membrane potential.** The cultured cells were incubated with 50 nM of TMRE (Abcam, Cambridge, MA) in medium for 30 mins, rinsed once with PBS, and viewed in PBS. The images were acquired by laser confocal microscope. The fluorescence intensity was analyzed by the software Image Pro-Plus.

**Measurement of reactive oxygen species (ROS)**. Cells were cultured with medium containing 25 μM 2’,7’-dichlorodihydro-fluorescein diacetate (DCFH-DA) (Abcam, Cambridge, MA) for 1 h, and then rinsed once with PBS and imaged by laser confocal microscope. The fluorescence intensity was analyzed by the software Image Pro-Plus. For analysis ROS levels in the hippocampus of APP/PS1 transgenic mice, the hippocampal lysates containing 0.5 mg protein were incubated with 10 μM DCFA-DA at 37 ºC for 60 mins. The fluorescence intensity was measured with excitation at 485 nm by a fluorescence microplate reader (Thermo Fisher Scientific, USA). The levels of ROS were presented as arbitrary fluorescence units (AFU).

**Analysis of colocalization of LC3 and mito-dsRed and fragmented mitochondria.** HeLa cells co-transfected with LC3-GFP and mito-dsRed plasmid were imaged with a confocal microscope. Colocalization ratio of LC3-GFP and mito-dsRed were analyzed by Image-Pro Plus 6.0 software (Media Cybemetics, Silver Spring, MD) as described ([Zinchuk & Grossenbacher-Zinchuk, 2009](#_ENREF_2)). After correcting the background, the Pearson's correlation coefficient was calculated as colocalization ratio. The mitochondrial fluorescent signals display as network or baculiform shape in normal condition. The cells contain fragmented mitochondria were defined as the cells contain mitochondrial signals in punctate shape. The percentage of cells with fragmented mitochondria was counted.

[**Immunofluorescence**](javascript:void(0);)**quantification.** [Immunofluorescence](javascript:void(0);) staining and quantification was performed as described ([Deng et al., 2016](#_ENREF_1)). The images were converted into 8-bit images, and binarized after subtracting the background noise, using NIH Image J software. Mean fluorescence intensities (MFIs) of SYN and lysenin was measured and calculated by dividing the MFI units by the area of outlined regions. The intensity threshold was set and kept constant for all images analyzed. The size of Aβ plaques was quantified as the areas of Aβ plaques by the total areas of the cortex or hippocampus. For quantification, the sections from five mice per group and at least eight hippocampal sections per mouse were analyzed.

**Transmission Electron Microscope (TEM).** Cells were fixed for 1 h in Karnovsky’s fixative (3% paraformaldehyde, 2% glutaraldehyde, 5 mM CaCl2 in 0.1 M cacodylate buffer [pH 7.4], containing 0.1 M sucrose). and embedded with agar noble to a final concentration of 1.7% and post fixed with 1% OsO4, 0.5% potassium dichromate, and 0.5% potassium hexacyanoferrate in 0.1 M cacodylate buffer. The pellet was stained en bloc with 2% aqueous uranyl acetate followed by ethanol dehydration and embedded in EMbed (EMS). Sections (75 nm) were cut, stained with 2% uranyl acetate in 50% ethanol and lead citrate, and examined using a FEI CM12 EINDHOVEN transmission electron microscope at an accelerating voltage of 120 kV. Digital images were obtained with MegaView3 CCD camera (SIS GMBH).

**Morris water maze.** Mice were taken 4 trials per day. A different starting position was used on each trial. The duration of a trial was 90 seconds. Escape latencies (time spent swimming from start point to the target) and path length (the distance from start point to the platform) before reaching the platform were recorded for 5 consecutive days. For probe trials, the platform was removed after the last trial of the acquisition period. The mice were tested 24 hours later to assess memory consolidation. The time spent in the target quadrant within 60 seconds was recorded. The latency to the first target site was measured, and the numbers of platform-site crossovers were recorded.

**Novel object recognition.** Mice were exposed to two identical objects for 10 min placed in two opposite corners of the apparatus from the sidewall. Ninety minutes after the training session, the animal explored the open field for 10 min in the presence of one familiar and one novel objects. Location preference = time exploring one of the identical objects/time exploring the identical object pairs × 100%. Recognition index (RI) = time exploring novel object/ (time exploring novel object + time exploring familiar object) × 100%.

**References**

Deng, Q.-S., Dong, X.-Y., Wu, H., Wang, W., Wang, Z.-T., Zhu, J.-W., . . . Schachner, M. (2016). Disrupted-in-Schizophrenia-1 Attenuates Amyloid-β Generation and Cognitive Deficits in APP/PS1 Transgenic Mice by Reduction of β-Site APP-Cleaving Enzyme 1 Levels. *Neuropsychopharmacology, 41*(2), 440.

Zinchuk, V., & Grossenbacher-Zinchuk, O. (2009). Recent advances in quantitative colocalization analysis: focus on neuroscience. *Prog Histochem Cytochem, 44*(3), 125-172. doi:10.1016/j.proghi.2009.03.001
